# Supplementary material for: The Influence of the COVID-19 Pandemic in NK Cell Subpopulations from CML Patients Enrolled in the Argentina Stop Trial
Source: Cells. 2025 Apr 23;14(9):628. doi: 10.3390/cells14090628 (PMC12072037; doi:10.3390/cells14090628)
Supplement: Supplementary file 1 [file cells-14-00628-s001.zip › cells-3524547-supplementary.pdf]

Variable (AST I vs AST II)	Mann-Whitney p-value	Corrected p-value (FDR)	
%NK	0.0051	0.0092	**
%NK CD57$^+$	0.0033	0.0066	**
%NK CD16$^+$	0.0222	0.0275	*
%NK NKp46$^+$	0.0081	0.0115	*
%NK NKp44$^+$	<0.0001	0.0002	***
%NK PD1$^+$	<0.0001	0.0002	***
%CD56^{dim}NKG2C$^+$CD57$^+$NKp30$^+$	0.0234	0.0275	*
%CD56^{dim}NKG2C$^+$CD57$^+$NKp30$^+$NKp46$^+$	0.0264	0.0293	*
%CD56^{dim}NKG2C$^+$CD57$^+$NKp46$^+$	0.0001	0.0002	***
%CD56^{dim}NKG2C$^+$NKp46$^+$	0.0061	0.0093	**
%CD56^{dim}CD107a$^+$	0.0571	0.0601	ns
%CD56^{dim}IFNy$^+$	0.002	0.0044	**
%CD56^{bright}IFNy$^+$	0.0058	0.0093	**
MFI CD107 in CD56^{dim}	<0.0001	0.0002	***
MFI IFNy in CD56^{dim}	<0.0001	0.0002	***
MFI IFNy in CD56^{bright}	<0.0001	0.0002	***
%CD107 in NKG2C$^+$NKp46$^+$ (%CD56^{dim})	0.0138	0.0184	*
%IFNy in NKG2C$^+$NKp46$^+$ (%CD56^{dim})	<0.0001	0.0002	***
HCMV titer	0.3168	0.3168	ns
SARS-CoV-2 titer	<0.0001	0.0002	***

Supplementary Table 1. Corrected p-values from comparisons between both patient cohorts.

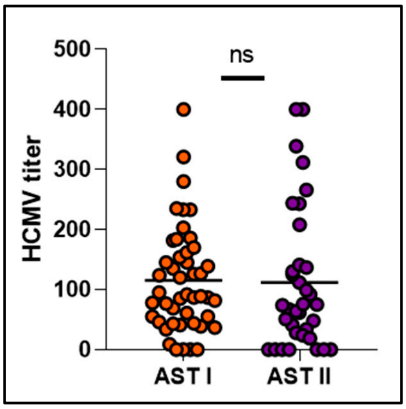

Supplementary Figure 1. Comparison of HCMV titer between both patient cohorts. Mann-Whitney test was performed, and p-values were corrected with the FDR method.
